# 12-week melatonin administration had no effect on diabetes risk markers and fat intake in overweight women night workers

**DOI:** 10.3389/fnut.2024.1285398

**Published:** 2024-01-22

**Authors:** Carlos Alberto Rodrigues de Sousa, Luciana Fidalgo Nogueira, José Cipolla-Neto, Claudia Roberta de Castro Moreno, Elaine Cristina Marqueze

**Affiliations:** ^1^Carlos Chagas Filho Biophysics Institute, Federal University of Rio de Janeiro, Rio de Janeiro, Brazil; ^2^Department of Epidemiology, Postgraduate Program in Public Health, Catholic University of Santos, São Paulo, Brazil; ^3^Department of Physiology and Biophysics, Institute of Biomedical Sciences, University of São Paulo, São Paulo, Brazil; ^4^Department of Health, Life Cycles and Society, Faculty of Public Health, University of São Paulo, São Paulo, Brazil

**Keywords:** fat, melatonin, circadian misalignment, night work, diabetes

## Abstract

**Introduction:**

Interactions between circadian clocks and key mediators of chronic low-grade inflammation associated with fat consumption may be important in maintaining metabolic homeostasis and may pose a risk for the development of obesity-associated comorbidities, especially type 2 diabetes (T2DM).

**Objective:**

The aims of the present study were to evaluate the effects of melatonin administration on diabetes risk markers according to dietary lipid profile (pro-inflammatory versus anti-inflammatory) in excessive weight night workers, and to determine the effect of administration on fat consumption profile.

**Methods:**

A randomized, controlled, double-blind, crossover clinical trial involving 27 nursing professionals working permanent night shifts under a 12×36-hour system. The melatonin group (12 weeks) used synthetic melatonin (3 mg) only on days off and between shifts, while the placebo group (12 weeks) was instructed to take a placebo, also on days off and between shifts. For inflammatory characteristics, participants were divided into pro-inflammatory (saturated fats, trans fats and cholesterol) and anti-inflammatory (monounsaturated, polyunsaturated fats and EPA + DHA) groups according to fatty acid determinations. At baseline and at the end of each phase, blood glucose, insulin, glycosylated hemoglobin plasma concentrations were collected, and HOMA-IR was calculated.

**Conclusion:**

Melatonin administration for 12 weeks had no effect on T2DM risk markers according to dietary lipid profile (pro-inflammatory or anti-inflammatory potential) in excessive weight night workers. Among the limitations of the study include the fact that the low dose may have influenced the results expected in the hypothesis, and individual adaptations to night work were not evaluated. The insights discussed are important for future research investigating the influence of melatonin and fats considered anti- or pro-inflammatory on glucose and insulin homeostasis related to night work.

## 1 Introduction

Engaging in night shift work can impact biological rhythms and is often associated with alterations in sleep patterns, poor quality of life and reduced recovery (1). Circadian misalignment at eating times, as well as activity during the night among night workers, has been associated with an increased risk of developing diabetes mellitus (DM), hypertension, obesity and cardiovascular disease (1–4).

Diet composition, especially a high-fat and high saturated fat (SFA) diet, modulates the rhythmicity of the peripheral circadian clock *in vitro* and *in vivo* (5, 6). There is evidence of an increased preference for high-fat foods for breakfast (6), where this is because the timing of this meal is a potential zeitgeber, promoting interactions between the body’s central clock and nutrient sensory pathways (e.g., AMPK). In this context, highly palatable foods, such as fats, can directly signal the orexigenic centers and regions associated with hedonic stimulation, stimulating food-seeking behavior (7–10).

Some studies have identified a higher fat intake among night workers. Heath et al. (11) found that fat intake by night workers exceeded the recommended level (with fat representing 20%–35% of total energy intake and comprising 15.5% saturated fat). In another study, of airline employees at high risk of developing type 2 diabetes mellitus (T2DM) and/or diabetics, the authors found that women over 47.6 years of age who worked day and night shifts on board (flight), had higher energy intake from fat (at 33.9%, comprising 12.8% saturated fat) compared to women in the same age group who worked exclusively during the day (12).

The dysmetabolism seen in night workers can lead to changes in eating patterns or vice versa, such as an increase in daily energy consumption (13), greater hunger and longer duration of food intake (14). Excess adiposity, now recognized as a low-grade inflammatory state, can also reduce insulin responsiveness in insulin-sensitive tissues and promote the risk of T2DM through action on circulating cells (15). In addition, insufficient and poor sleep quality, often associated with night work, is also associated with a higher likelihood of obesity and DM, further increasing the risk of this group of workers (1, 16, 17). In situations of low-grade inflammation, such as obesity, the chronicity of in-flammation can lead to comorbidities, such as cardiovascular disease, insulin resistance, anemia, hyperlipidemia, metabolic syndrome, T2DM and cancer (18–20). Among the promising therapeutic strategies, it was recently demonstrated that melatonin, as the main product of the pineal gland, is considered a broad-spectrum antioxidant, can be applied in pathological conditions such as T2DM, mainly for its regulatory effects on the expression of the glucose transporter gene type 4 (GLUT4), glucose homeostasis and insulin sensitivity (21–23).

Another risk factor for night workers is a decrease in melatonin levels caused by exposure to artificial light during the work shift, a phenomenon associated with the risk of developing T2DM (24–28). The role of melatonin is mainly related to biological rhythms and the coordination of behavioral and physiological adaptations to the light–dark cycle, i.e., the hormone acts as an important regulator of allostasis (29). Cipolla-Neto et al. (30) suggested that supplementation or replacement could improve metabolic changes associated with reductions in serum melatonin levels, as occurs among night workers. Regarding glucose metabolism, a meta-analysis of 12 clinical trials showed that melatonin administration reduced fasting blood glucose, but had no influence on levels of glycated hemoglobin, insulin or insulin resistance (IR) (31). Subsequently, another meta-analysis with 16 studies showed positive results for the administration of melatonin on glucose metabolism, with a dose ranging between 3 and 10 mg for up to 24 weeks of duration (32).

Melatonin administration, given its action on mechanisms of glycemic homeostasis, inflammation, and energy metabolism (33–35), can represent a therapeutic and/or preventive alternative for metabolic alterations associated with night work. Recent research has demonstrated that the administration of 3 mg of melatonin in overweight individuals reduced circadian misalignment, especially among the most misaligned (earlier chronotype) and reduced body weight and body mass index (BMI), without changing caloric intake or participants’ physical activity levels (36, 37). Animal models supplemented with melatonin and subjected to circadian misalignment associated with a high-fat diet showed significant improvement in fasting glucose, oral glucose tolerance and inflammatory profile (38, 39). However, no studies investigating the effect of melatonin administration on DM markers based on dietary lipid profile have been conducted in humans, specifically among night workers.

Thus, the aim of the present study was to evaluate the effects of melatonin administration on DM risk markers (glucose, insulin, glycosylated hemoglobin and HOMA-IR), according to dietary lipid profile (pro-inflammatory versus anti-inflammatory) in excessive weight night workers. Therefore, the study hypothesis holds that melatonin administration improves diabetes risk markers in excessive weight night workers who have a diet of predominantly anti-inflammatory fats, and also decreases fat consumption.

## 2 Manuscript formatting

### 2.1 Study type

The present study is a randomized, controlled, double-blind, crossover clinical trial, evaluating the effect of melatonin on dietary lipid consumption and its influence on diabetes markers in night workers. The present project is part of a larger study, detailed in-formation on the original study is available from Marqueze et al. (36).

### 2.2 Population and sample

The participants of the present study were nursing professionals (nurses and nursing technicians) who worked permanent night shifts under a 12×36-hour system (12 h on, 36 h off) at a large private hospital in São Paulo, Brazil. The sample power was calculated *a posteriori* based on the test of difference of repeated measures (within-between interaction), an effect size of 0.25, alpha error of 5%, two groups (consumption and intervention), and three measures (baseline, melatonin, and placebo). Thus, the study population of 27 participants had a sample power of 80% (G*Power).

### 2.3 Inclusion and exclusion criteria

Subjects that met the following criteria were included in the study: women; aged 20–50 years; body mass index (BMI) ≥25 kg kg/m^2^ and < 40 kg/m^2^; working night shift for ≥6 months; who declared having no intention of following any restricted diets and starting new physical activities while participating in the study. Individuals that met the following criteria were excluded from the study: women who were pregnant, nursing, had infants aged <1 year; were experiencing the climacteric or menopause period; held a second night job; were in regular use of medications or dietary supplements that influence sleep, alertness or the circadian timing system (barbiturates, antidepressants, benzodiazepines, melatonin, ritalin, modafinil, sleep aids); had a past history of neurological or psychiatric illnesses, or drug and alcohol abuse; circadian or sleep disorders; presented metabolic problems (except participants with T2DM and treated dyslipidemias or using statins and anti-triglycerides); cardiovascular diseases (except treated systemic arterial hypertension); clinically-diagnosed inflammation and/or chronic infections; eating disorders (bulimia, anorexia); had anemia or had donated >400 mL of blood in the three months preceding the study; had undergone major surgery in the last six months prior to participating in the research.

### 2.4 Data collection and data processing

Initially, the human resources sector of the institution was contacted and provided a list of all nursing professionals. A total of 238 female professionals engaged in permanent night shifts under the 12×36-hour system were identified. Shortly after this process, the nursing professionals were contacted, informed about the study, and invited to undergo screening according to the study inclusion and exclusion criteria.

Pre-screening took place from February to April 2018 and was carried out individually at participants´ workplace during their shift. Those professionals who met the inclusion and exclusion criteria (*n* = 46) were invited to participate in the survey and specific dates were scheduled for baseline data collection.

The clinical trial was conducted from April 2018 to August 2019. It is important to mention that seasonality was not a limiting factor in the present study, given that the length of days and nights are almost equivalent in Brazil, resulting in a photoperiod close to 12 h per day (40). After the collection of initial data (baseline), participants were randomized into two groups, with 23 women allocated to the first group (Melatonin Group – GM) and 23 to the second group (Placebo Group – PG) for a 12-week period. In the second phase of this crossover study (lasting three months), volunteers allocated to the intervention group in the first phase switched over to the control group for the second phase and vice versa (control group subjects switched over to intervention group). In the second phase, 19 volunteers (41.3%) discontinued the study during the protocol because they had started a second night job, became pregnant, changed shifts, or quit their job. Although we did not perform intention-to-treat analysis, which may be a limitation of the study, our final sample met the number necessary for good sampling power (80%).

Sociodemographic characteristics, along with work and health-related aspects, were collected through self-administered questionnaires. Although questionnaires were completed by participants, a researcher was always available to clarify any doubts. All assessments took place at the participants´ workplace during their working hours, between 12:00 am and 05:00 am, according to the shift schedule provided by the institution. Assessments took place at baseline, in the last 10 days of the first phase, and in the last 10 days of the second phase, including the evaluation of biochemical parameters.

### 2.5 Protocol

Each participant followed the protocol for 25 weeks (12 weeks for intervention, 12 weeks for placebo, plus 1 week for baseline). The GM group used synthetic melatonin only on days off and between shifts, i.e., on days when they slept during the night. On night workdays, melatonin was not taken by the participants. All participants were instructed to take a fast-release 3 mg melatonin tablet (Aché Pharmaceutics^®^, Brazil), one hour before the desired time to go to sleep. Subjects filled out a diary with information on the time they took melatonin, as well as bedtime and waking times. It is important to mention that the melatonin administration has not been associated with adverse events to date; however, the volunteers were instructed to report any symptoms to the researchers so that the necessary referrals could be made, but no symptoms or discomfort were reported by the participants.

**Figure 1:**
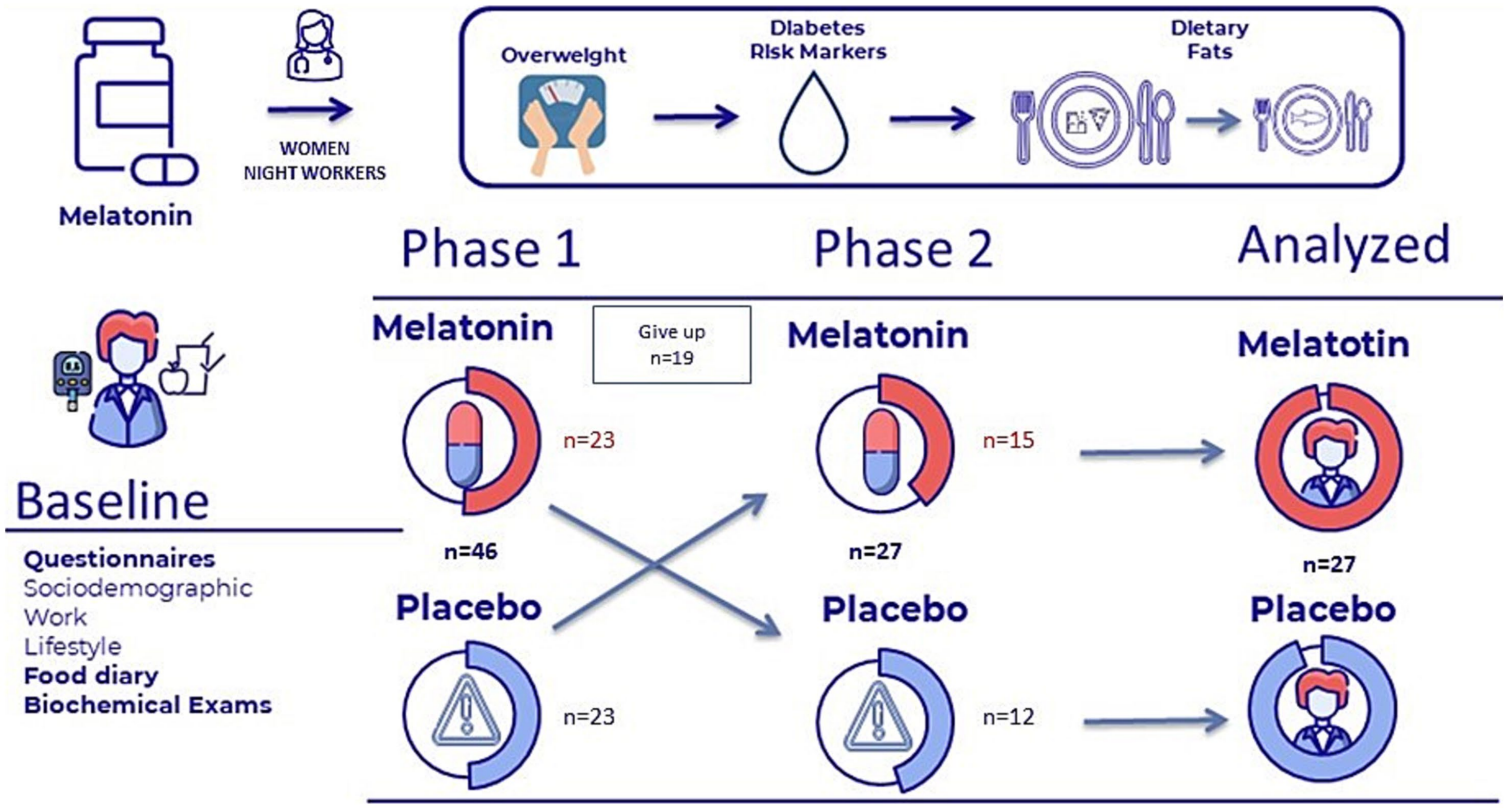
Illustrated diagram of the study.

The PG group was instructed to take a placebo pill identical in appearance to the melatonin pill, receiving the same instructions for use as the intervention group. The placebo pill resembled melatonin but contained no active ingredient, exerting no effect on the body (gluten-free and lactose-free). The study was double-blind, where neither the participants nor the lead researcher was aware of whether the study subjects were part of the intervention group or the control group. Over the three months of each phase, participants took melatonin for an average of 45 days (SD 10.3 days) and placebo for 44.3 days (SD 8.2 days). The length of the study was 18 months, as not all participants started the protocol at the same time. The illustrated diagram of the study is presented in Figure 1. Marqueze et al. (36) present detailed information about the study protocol.

### 2.6 Dietary assessment

Monthly, study participants kept food diaries on a typical working day and a typical day off (for a total period of 7 months, with the first evaluation completed before commencement of the protocol). The time window for recording this data was from 7:00 pm to 7:00 pm the following day, for both working days and days off. Food diaries are a good method for assessing dietary patterns (41), and one-day records have previously been used in other studies (42, 43). The evaluations were conducted by a duly trained nutritionist, under the supervision of the research coordinator. It is important to mention that the assessment of the dietary pattern (one working day and one day off) was first performed at baseline and again for every month of the protocol thereafter (at three-time points), totaling seven months and 14 days of food records. Importantly, participants had the option of a nutritionist-planned dinner provided by the hospital. In addition, all units had a pantry where they could store food brought in from outside the hospital and have meals.

The diaries were analyzed using the Nutrition Data System for Research (NDSR – United States Department of Agriculture – USDA), 2007 version. Due to cultural differences between Brazilian and North American food consumption (with the US serving as the reference for the NDSR), the composition of typical Brazilian foods and preparations was added based on the Brazilian Food Composition Table (44) and on specific processed food labels. Only two participants reported using dietary supplements, which were included in the assessment of food consumption, and there were no significant monthly differences in food intake.

The dietary lipid profile was determined based on saturated, monounsaturated and polyunsaturated fats, trans fats, cholesterol, eicosapentaenoic acid (EPA) + docosahexaenoic acid (DHA) and total fat (grams). The evaluation of the dietary lipid profile regarding DM markers was based on total fat (≥ 35% E) (45). The dietary fatty acid profile, according to inflammatory status, was attributed by tallying the average intake (grams) of dietary lipid recorded for working days and days off over the study period, i.e., at baseline and the end of the first and second phases (3 timepoints) and grouping these according to the following classification (46, 47).

1. Pro-inflammatories = saturated fats, trans fats and cholesterol;

2. Anti-inflammatories = monounsaturated and polyunsaturated fats, eico-spentaenoic acid (EPA) + docosahexaenoic acid (DHA).

The adequacy of the dietary pattern was estimated using the Dietary Reference Intakes (DRI) and in cases where these were not available, the Recommended Dietary Allowances (RDA), both established by the US National Academy of Sciences (45). Dietary profile was established based on: 1. Total caloric intake (kcal/day); 2. Carbohydrates (45-65%E); 3. Fats (20-35%E); 4. Proteins (10-35%E); and 5. Dietary fiber (14 g/1000 kcal) (45). For fat profile, the recommendations of the updated Brazilian Directive on Dyslipidemia and Prevention of Atherosclerosis – 2017 were used (48).

### 2.7 Risk markers for diabetes

For determination of plasma concentrations of blood glucose, insulin, glycosylated hemoglobin and HOMA-IR (Homeostasis Model Assessment of Insulin Resistance), blood collection was performed after a 12-h fast, before morning medication, with no consumption of alcoholic beverages the day before collection, and after a night’s sleep of at least six hours after the volunteers’ days off. Glycemic profile values were evaluated according to the criteria of the American Diabetes Association (49). The variables include the following criteria: blood glucose (≥100 mg/dL), insulin (≥23 ng/dL), glycosylated hemoglobin (≥5.7%) and HOMA-IR. The blood glucose and insulin values provided the basis for calculating the HOMA-IR index, using the formula: HOMA-IR = fasting glucose in mmol/l x fasting insulin in μU/mL/22.5 (50). According to the BRAMS study, a value of >2.71 was adopt-ed to identify IR in adults (51).

### 2.8 Study variables

The dependent variables of the study were the diabetes risk markers (glycemia, insulin, glycosylated hemoglobin and the HOMA-IR index) and the profile of dietary lipids (saturated, monounsaturated, polyunsaturated, trans, cholesterol, EPA + DHA) consumed by the participants during the intervention. The independent variable was the inflammatory profile of dietary lipids (pro-inflammatory or anti-inflammatory). The adjustment variables included age, BMI and total time working nights. The descriptive variables were age, education, job role at hospital, weekly working hours (hours/week), net income, smoking status, weekly physical activity time (where ≥150 min of moderate or intense leisure-time activity per week was defined as physically active) (52, 53), dietary profile (described in dietary assessment) and anthropometric data (weight and height) measured according to Lohman et al. (54). Body Mass Index (BMI) in kg/m^2^ was calculated from the measurements of weight (kg) and height (m). For the classification of BMI for adults, the criterion recommended by the WHO was adopted, where a value >24.99 kg/m^2^ indicates overweight (55).

### 2.9 Statistical analysis

The Shapiro–Wilk test was applied to test the normality of the quantitative variables. Parametric variables were expressed as mean and standard deviation (SD) or standard error (SE), while non-parametric variables were expressed as median and interquartile range-IQR (P25%–P75%). The Wilcoxon test was used to observe differences in fat consumption on days off and on working days.

The effects of melatonin administration on dietary lipid profile and DM markers according to the inflammatory profile of dietary lipids was assessed using a generalized linear model (GLM) with the LSD (Least Significant Difference) *post hoc* test for the test comparing 3 means (related samples) with two factors (intervention and dietary lipid pro-file). In all tests, a *p*-value <0.05 was considered significant. All data was analyzed using Statistica 12.0 and STATA 14.0 (Stata corp, Texas, United States) packages.

### 2.10 Ethical aspects

The project was approved by the Research Ethics Committee of the School of Public Health of the University of São Paulo (FSP-USP) (protocol no 2,450,682, December 20, 2017) and by the Ethics Board of the participating Hospital (protocol No. 2,489,636, February 7, 2018). The study was registered with the Brazilian Registry of Clinical Trials (RBR-6pncm9) and on the International Clinical Trials Registry Platform of the World Health Organization (UTN no U1111-1238-7395). Participants were guaranteed confidentiality and anonymity, and the study was only carried out after participants had a clear understanding of the objectives of the study and signed the Free and Informed Consent Form, which complied with Resolution 466/2012.

## 3 Results

At the end of data collection period, 27 volunteers had completed the intervention. Participants had a mean age of 37.1 years (SD 5.9 years, IQR 32.1–42.6 years) and mean BMI of 29.9 kg/m^2^ (SD 3.3 kg/m^2^), fifteen were overweight and twelve were obese. Mean time working at the hospital was 8.4 years (SD 4.4 years) and the median time working nights at the institution was 5.3 years (IQR 2–4 years). Most participants had attained postgraduate education, and more than half had an income >5,000 Brazilian reais (BRL) per month. For marital status, most participants reported having a partner (Table 1).

**Table 1:** Sociodemographic data of participants (*n* = 27).

Variables	*n*	%
**Education**		
High school	6	22.2
College incomplete or studying	5	18.5
Incomplete or ongoing postgraduate studies	5	18.5
Complete postgraduate studies	11	40.8
**Family income (BRL month)**		
1,001–3,000	1	3.7
3,001–5,000	7	25.9
5,001–10,000	15	55.6
>10,000	4	14.8
**Marital status**		
No partner	8	37.0
Partner	17	63.0
**Reason for working at night**		
Required by service	1	3.7
Reconcile with another job	1	3.7
Reconcile with home care	11	40.7
Affinity for job	5	18.5
Supplement income	8	29.6
Do not know/Do not remember	1	3.7
**Physical activity**		
Active	9	34.6
Sedentary	17	65.4

Regarding the reasons for working nights, most volunteers chose this shift to reconcile work with home care. A sedentary lifestyle (< 150 min/week of moderate physical activity) was predominant among the study participants.

Participants had an average energy consumption of more than 1,500 kcal/day, and the distribution of macronutrients (carbohydrates, proteins, and lipids) was within recommended values. Regarding dietary fiber, the volunteers had low consumption at baseline. Mean dietary cholesterol intake was within recommended levels (Table 2).

**Table 2:** Dietary pattern (average days off plus days at work) and diabetes markers at baseline of participants (*n* = 27).

**Variables**	Mean ± SD or Median [IQR]	Reference values
Energy (kcal)	1,575.2 [1,177.2–2,057.6]	
**Macronutrients**		
Carbohydrates (%E)	52.2 ± 8.1	45–65%
Proteins (%E)	17.8 ± 4.1	10–25%
Total fat (%E)	29.4 ± 6.7	20–35%
Fibers (g)	15.1 [12.5–12.5]	14 g/1,000 kcal
Cholesterol (g)	194.49 [108.7–311.5]	<300 mg/day
**Breakdown of fatty acid intake**		
Saturated (%E)	10.3 ± 3.6	<10%
Trans (g)	1.4 [0.8–2.7]	Exclude from diet
Monounsaturated (%E)	9.7 ± 2.9	15%
Polyunsaturated (%E)	6.7 ± 2.1	5–10%
DHA + EPA (g)	0.035 [0.022–0.085]	> 0.5 g
**Diabetes markers**		
Glucose (mg/dl)	95.6 ± 9.0	≥100 mg/ dL
Insulin (μU/ml)	14 [8–20]	≥23 μU/ml
HbA1c (%)	5.3 ± 0.4	≥5.7%
HOMA –IR	3.1 [2.0–4.8]	>2.71

%E, percentage of energy; g = grams; mg = milligram; μg = microgram; [], Interquartile range (IQR); ±, Standard deviation (SD); HbA1c, glycated hemoglobin; HOMA-IR, homeostasis model assessment of insulin resistance.

Mean values for glycemia, insulin, and HbA1c were all within recommended reference ranges, but the mean HOMA-IR index was high (Table 2), and the presence of IR at baseline was detected in 17 (62.9%) volunteers. While none of the participants had values consistent with DM at baseline, 9 (33.3%) participants had glycemia indicative of pre-diabetes (≥100 mg/ dL).

Regarding total consumption of fats during the study, consumption levels of 81.5% of participants were within recommended ranges (20-35%E), with a median consumption of 56.4 g/day of fat (IQR 45.0–66.9 g/day). Notably, only four individuals had a high consumption of total fat (≥35%E) during the study period, but consumption of both saturated and trans fatty acids was high, whereas the intake of long-chain monounsaturated and polyunsaturated fatty acids was low (DHA + EPA) (Table 3).

**Table 3:** Fat profile (mean of baseline, melatonin and placebo) on days off and work days of participants during study (*n* = 27).

	Work days	Days off	
Fats	Mean ± SD or median [IQR]	Mean ± SD or median [IQR]	p*
Saturated (g)	19.9 [12.4–23.8]	20.9 [14.6–24.9]	0.27
Trans (g)	2.1 [1.1–2.3]	2.4 [1.1–2.5]	0.02
Cholesterol (g)	0.12 [0.07–0.15]	0.14 [0.07–0.2]	0.92
Pro-inflammatory (total)	21.3 [13.6–26.0]	23.5 [16.5–27.2]	0.14
Polyunsaturated (g)	12.5 [7.8–15.8]	12.2 ± 4.3	0.89
Monounsaturated (g)	19.3 [12.1–22.8]	19.6 ± 6.4	0.72
DHA + EPA (g)	0.21 [0.03–0.3]	0.17 [0.02–0.20]	0.25
Anti-inflammatory (total)	31.9 [20.9–42.1]	31.9 ± 9.9	0.85

* Wilcoxon test. ±, Standard deviation (SD); g = grams; [], Interquartile range (IQR). DHA + EPA, eicospentaenoic acid + docosahexaenoic acid.

The profile of fats consumed at each of the three timepoints is shown in Table 4. Results show that melatonin administration had no effect on the individual intake profile of each fat (saturated, trans, polyunsaturated, monounsaturated, EPA + DHA and cholesterol). For this evaluation, we chose not to separate consumption on days off and work because, as previously tested, we observed statistical differences only for saturated fat. Likewise, no effect on consumption was evident when grouped according to inflammatory characteristics or total fat (g).

**Table 4:** Effect of melatonin administration on dietary lipid consumption profile of participants (*n* = 27)*.

Fats	Baseline	Melatonin	Placebo	p**
Saturated (g)	21.10 ± 2.81	19.35 ± 1.24	19.60 ± 1.22	0.26
Trans (g)	1.55 ± 0.31	2.23 ± 0.18	2.46 ± 0.30	0.90
Cholesterol (mg)	224.80 ± 29.34	76.58 ± 9.95	101.20 ± 13.15	0.50
Pro-inflammatory (g/total)	22.86 ± 3.09	21.65 ± 1.31	22.14 ± 1.35	0.32
Polyunsaturated (g)	13.03 ± 1.45	11.72 ± 0.80	12.23 ± 1.01	0.52
Monounsaturated (g)	19.54 ± 2.28	19.23 ± 1.17	19.53 ± 1.37	0.62
EPA + DHA (g)	0.18 ± 0.07	0.18 ± 0.05	0.21 ± 0.05	0.21
Anti-inflammatory (g/total)	32.75 ± 3.43	31.12 ± 1.89	31.99 ± 2.29	0.57
Total (g)	58.92 ± 6.51	55.50 ± 3.24	56.44 ± 3.66	0.47

* Model adjusted for age, BMI and total time working nights. ** Generalized Linear Model. ±, standard error. DHA + EPA, eicospentaenoic acid + docosahexaenoic acid.

Melatonin administration alone exerted no influence on the biochemical markers evaluated. Similarly, no effects of melatonin administration on level of total fat consumption, were found (Table 5). The assessment of fats dichotomized into pro-inflammatory and anti-inflammatory groups also showed no isolated effect on glycemic parameters or interaction with exogenous melatonin (Table 6).

**Table 5:** Effect of melatonin on diabetes markers, according to consumption pattern, during intervention (mean baseline, melatonin and placebo) for total fats (%E) in excessive weight night workers (*n* = 27)*.

	Baseline	Melatonin	Placebo	Baseline	Melatonin	Placebo	Consumption	Intervention	Consumption × Intervention
	Mean (SE)	Mean (SE)	Mean (SE)	Mean (SE)	Mean (SE)	Mean (SE)	p**		
% Energy	≥35% (*n* = 4)			<35% (*n* = 23)					
Glucose	96.0 (1.7)	94.7 (1.7)	95.6 (1.8)	89.7 (4.1)	90.0 (4.2)	87.0 (4.3)	0.36	0.08	0.24
HbA1c	5.3 (0.1)	5.3 (0.1)	5.4 (0.1)	5.2 (0.2)	5.5 (0.2)	5.3 (0.2)	0.79	0.17	0.49
Insulin	15.4 (1.4)	13.57 (1.1)	16.4 (2.7)	10.0 (3.4)	10.8 (2.6)	15.0 (6.4)	0.67	0.63	0.95
HOMA-IR	3.7 (0.4)	3.20 (0.4)	3.9 (0.6)	2.2 (0.9)	2.4 (0.6)	3.2 (1.5)	0.50	0.57	0.98

*Model adjusted for age, total time working nights and BMI. **p-values calculated by generalized linear model (GLM). *p* < 0.05 was considered significant. SE, standard error. HbA1c, glycated hemoglobin; HOMA-IR, homeostasis model assessment of insulin resistance.

**Table 6:** Effect of melatonin on diabetes markers, according to consumption pattern, during intervention (mean baseline, melatonin and placebo) for pro-inflammatory (g) and anti-inflammatory (g) fats in excessive weight night workers (*n* = 27)*.

	Baseline	Melatonin	Placebo	Baseline	Melatonin	Placebo	Consumption	Intervention	Consumption × Intervention
	Mean (SE)	Mean (SE)	Mean (SE)	Mean (SE)	Mean (SE)	Mean (SE)	p**		
Pro-inflammatory	Lower consumption (*n* = 13)			Higher consumption (*n* = 14)					
Glucose	98.6 (2.3)	96.1 (2.3)	96.7 (2.5)	92.1 (2.2)	92.1 (2.2)	92.1 (2.4)	0.93	0.22	0.91
HbA1c	5.4 (0.1)	5.4 (0.1)	5.4 (0.1)	5.2 (0.1)	5.4 (0.1)	5.4 (0.1)	0.83	0.23	0.06
Insulin	16.5 (1.9)	13.3 (1.4)	13.6 (3.4)	12.9 (1.8)	13.0 (1.4)	18.5 (3.3)	0.26	0.84	0.29
HOMA-IR	4.0 (0.5)	3.1 (0.3)	3.9 (0.8)	2.97 (0.5)	3.0 (0.3)	4.3 (0.8)	0.30	0.92	0.26
Anti-inflammatory	Lower consumption (*n* = 14)			Higher consumption (*n* = 13)					
Glucose	92.6 (2.2)	92.1 (2.2)	90.9 (2.3)	97.7 (2.3)	96.1 (2.3)	98.0 (2.4)	0.39	0.11	0.40
HbA1c	5.2 (0.1)	5.5 (0.1)	5.4 (0.1)	5.34 (0.1)	5.4 (0.1)	5.3 (0.1)	0.59	0.15	0.37
Insulin	15.1 (1.7)	14.0 (1.4)	18.6 (3.3)	14.1 (1.8)	12.2 (1.4)	13.5 (3.4)	0.11	0.60	0.81
HOMA-IR	3.5 (0.5)	3.2 (0.3)	4.2 (0.8)	3.4 (0.5)	2.9 (0.4)	3.4 (0.8)	0.20	0.57	0.97

*p-values calculated by generalized linear model (GLM). *p* < 0.05 was considered significant. **Model adjusted for age, total time working nights and BMI. SE, standard error. HbA1c, glycated hemoglobin; HOMA-IR, homeostasis model assessment of insulin resistance.

## 4 Discussion

In the present study, melatonin administration promoted no improvement in risk markers for diabetes, according to the inflammatory profile of dietary lipids in excessive weight night workers and exerted no effect on dietary lipid profile. Some studies involving animal and human models have shown positive results after melatonin administration in terms of blood glucose and insulin resistance under various conditions (56–58). However, there is a dearth of clinical trials involving night workers. In animal models with female guinea pigs that received 10 mg/kg of melatonin and 45% dietary lipid and were exposed 24 h a day to artificial light, administration significantly improved oral glucose tolerance (39). In another study, of mice without circadian misalignment that received 60% fat and were treated with melatonin, showed reduced fasting blood glucose (38). However, results of the present study failed to replicate these findings, where chronic circadian misalignment may have attenuated the effect of the intervention on common markers of glycemic homeostasis.

In another study, employing the same dose of melatonin administered in the present study (3 mg), Modabbernia et al. (58) supplemented 36 schizophrenic women (mean age 33 years) for eight weeks and observed no subsequent improvement in DM parameters (fasting glucose, insulin and HOMA-IR). By contrast, a meta-analysis by Delpino, Figueiredo and Nunes (32), nine out of the 15 studies showed a beneficial effect of melatonin on DM markers. Of the studies included in the analysis, two evaluated women only, with results showing improvement in insulin resistance and fasting glucose.

In the present study, it is important to consider the chronic circadian misalignment presented by the volunteers, who exhibited insulin resistance, even though their glucose and glycated hemoglobin levels were within the reference range. These factors might have prevented improvement in glucose intolerance and insulin resistance after melatonin administration (59, 60). To explain this effect, at the molecular level in Rizza et al. (61), night workers have a high REV-ERBα/BMAL1 mRNA ratio (possible chronic circadian misalignment due to exposure to prolonged artificial light) associated with a significant correlation between HbA1c and the expression of IL-1β RNA in leukocytes, even with values of glycemic parameters within reference standards, very common in low chronic inflammation associated with the risk of T2DM (61).

Regarding total fat consumption in the present study, despite not reaching statistical significance, there was a tendency towards a reduction or maintenance of fasting glucose, insulin, glycosylated hemoglobin and HOMA-IR, after melatonin administration in the presence of adequate consumption of total fat in the diet. This same pattern, however, was not observed in participants with inadequate consumption. This suggests a possible improvement in subclinical glucose tolerance, suppressed by the inflammatory status caused by the chronic misalignment inherent to chronic night work (62, 63).

Given the importance of the composition of dietary lipids in the pathophysiology of chronic diseases, especially T2DM, in the present study, fats consumed were dichotomized according to the characteristics of inflammatory responses (pro-inflammatory or anti-inflammatory), a well elucidated theory in the literature (64–66). Given the knowledge that night workers tend to have a more pro-inflammatory diet, the influence of the inflammatory profile of the fats present in the diet on melatonin response in the present study was explored (67, 68). However, no significant difference was found after dichotomizing dietary lipids regarding the pro-inflammatory factor. Explaining this effect, SFAs make up most of the pro-inflammatory component, and SFA ingestion is known to cause more marked lipemia than MUFAs or PUFAs, which can lead to a higher pro-inflammatory state exacerbated by low-grade inflammation and circadian misalignment (69–72).

Similarly, the amount of fats considered anti-inflammatory in the study proved unable to modify risk markers for DM following melatonin administration, even though blood glucose, insulin and HOMA-IR values were generally lower after administration. Consistent with the current study results, an isocaloric or *ad libitum* anti-inflammatory diet rich in mono and polyunsaturated fats (37% or 113 g of fat), with a saturated fat and cholesterol content below recommended levels, in individuals with DM and/or or pre-diabetes and obesity without circadian misalignment, was associated with a reduction in fasting blood glucose levels yet had no effect for insulin (73). On the other hand, in a controlled human model, exogenous melatonin alone influenced insulin sensitivity after a high-fat meal, with or without exposure to artificial light (62). This may explain the IR marker with higher values for placebo under all conditions in the present study.

The dietary profile found in the present study exhibits characteristics previously reported in the literature, demonstrating that night workers have more pro-inflammatory eating patterns compared to day workers, suggesting this may increase the risk of chronic diseases related to inflammation, such as DM (11, 74, 75).

Regarding the composition of fatty acids in the diet, consumption of saturated fat (%E) and trans fats by the study participants exceeded recommended levels, while low consumption of monounsaturated fatty acids and EPA + DHA was also observed, a profile commonly reported in studies assessing dietary intake among night workers (11, 12, 76, 77). Toward explaining this phenomenon, in the presence of circadian misalignment, the most palatable foods such as fats, act as potential zeitgebers, having a rapid direct effect on the orexigenic centers and regions associated with hedonic stimulation/reward, influencing food-seeking behaviour (7–9, 78).

In this context, fluctuations in fat consumption occurred among the night workers assessed, regardless of their job role. Since the volunteers in the present study performed half of the working days of the month at night, this variation in food consumption can be very common. Thus, Hemiö et al. (76) demonstrated that, among women at risk for DM, despite adequate fat consumption (≤35%), an increase of just one night shift was associated with an increase in total fat and saturated fat intake. Recently, plasma markers of lipid and liver function were found to have endogenous circadian rhythms that changed in response to a combined light and isocaloric meal schedule (27%E) (79). Although the authors did not assess glycemic regulation, this result may reinforce the importance of the present study, given that a specific macronutrient associated with glycemic dysregulation was investigated.

From this perspective, the composition of fatty acids and cholesterol in the diet is influenced by food choices, which in turn may be associated with circadian misalignment, leading to a greater preference for high-fat foods after night work. However, there is still no robust evidence on the relationship of melatonin administration with reduction in food consumption (6, 80).

The present study has several noteworthy strengths, including the assessment of the dietary profile every month throughout the intervention, the work in permanent shifts, i.e., the number of nights was the same among the participants, and also the fact that, to date, this is the only study that evaluates the influence of melatonin administration on DM risk markers in a double-blind randomized clinical trial under real-life conditions. The limitations of the study include the fact that the low dose may have influenced the expected results in the hypothesis, and individual adaptations to night work, circulating melatonin, and sleep assessments were not evaluated, factors that may have influenced the results. Although the study hypotheses were not confirmed, the insights discussed are important for future research investigating the influence of melatonin and fats considered anti- or pro-inflammatory on glucose and insulin homeostasis related to night work.

## 5 Conclusion

In summary, melatonin administration for 12 weeks had no effect on DM risk markers according to dietary lipids profile (pro-inflammatory or anti-inflammatory potential) in excessive weight night workers. Regarding fat consumption, melatonin administration promoted no change in consumption profile throughout the intervention for total fats, dichotomized into anti-inflammatory or pro-inflammatory types, or for isolated fats (saturated fat, trans fat, cholesterol, monounsaturated fat and EPA + DHA).

In the present study, the total consumption of anti-inflammatory fats was higher than pro-inflammatory fats, although a high consumption of saturated and trans-fat was evident and, in parallel, a low intake of monounsaturated and EPA + DHA. Therefore, given the originality of the topic addressed in the present study, future studies should be encouraged that involve a longer administration time, individualized doses, and possibly concomitant dietary prescription.

## Data availability statement

The original contributions presented in the study are included in the article/supplementary material, further inquiries can be directed to the corresponding author.

## Ethics statement

The studies involving humans were approved by Research Ethics Committee of the School of Public Health of the University of São Paulo (FSP-USP) (protocol no 2,450,682, December 20, 2017) and by the Ethics Board of the participating Hospital (protocol no. 2,489,636, February 7, 2018). The studies were conducted in accordance with the local legislation and institutional requirements. The participants provided their written informed consent to participate in this study.

## Author contributions

CS: Conceptualization, Formal analysis, Investigation, Methodology, Visualization, Writing – original draft, Writing – review & editing. LN: Conceptualization, Methodology, Supervision, Writing – original draft, Writing – review & editing. JC-N: Conceptualization, Project administration, Validation, Visualization, Writing – review & editing. CM: Conceptualization, Methodology, Validation, Visualization, Writing – review & editing. EM: Conceptualization, Data curation, Formal analysis, Funding acquisition, Investigation, Methodology, Project administration, Resources, Supervision, Visualization, Writing – original draft, Writing – review & editing.
